# Thermodynamic and environmental assessment of apple production in Türkiye: regional comparison and agrivoltaic integration

**DOI:** 10.1038/s41598-025-31495-z

**Published:** 2025-12-05

**Authors:** Müjdat Öztürk, Ramazan Kayabaşi, Hasan Yildizhan, Arman Ameen

**Affiliations:** 1 Department of Mechanical Engineering, Faculty of Engineering and Architecture, Kırşehir Ahi Evran University, 40100 Kırşehir, Turkey; 2 Department of Construction, Tomarza Mustafa Akincioglu Vocational School, Kayseri University, 38940 Kayseri, Turkey; 3 Department of Energy Systems Engineering, Adana Alparslan Türkeş Science and Technology University, Adana, Turkey; 4 Department of Building Engineering, Energy Systems and Sustainability Science, University of Gävle, Gävle, 801 76 Sweden

**Keywords:** Energy and exergy analysis, Apple cultivation, Agrivoltaic systems, Thermodynamic sustainability, Environmental assessment, Climate sciences, Energy science and technology, Environmental sciences, Environmental social sciences

## Abstract

This study presents a comprehensive thermodynamic and environmental assessment of apple cultivation across three major production regions in Türkiye: Antalya, Isparta and Niğde. This study is the first to provide an integrated energy, exergy and environmental assessment of agricultural voltaic systems by conducting a resource efficiency and sustainability assessment for open field apple production in Türkiye. Using a functional unit of one ton of apple production, the analysis integrates cumulative energy consumption (CEnC), cumulative exergy consumption (CExC) and cumulative carbon dioxide emissions (CCO_2_E) to reveal the sustainability performance of regional farming systems. The results indicate significant spatial variations linked to climatic and operational factors. Niğde exhibited the highest total energy (3098 MJ/ton) and exergy (2975 MJ/ton) consumptions, mainly due to diesel-powered irrigation and mechanization, resulting in a cumulative carbon footprint of 125 kg CO_2/_ton. Conversely, Antalya recorded the lowest total emissions (33 kg CO_2/_ton) with a balanced energy profile dominated by fertilizers and electricity use. Isparta demonstrated the most thermodynamically efficient and renewable system, achieving the highest cumulative degree of perfection (CDP) (3.80) and Renewability Index (RI) (0.74) values. The integration of agrivoltaic systems (AVS) has further enhanced sustainability across all provinces, particularly in Niğde, by increasing CDP by up to 97%. These findings highlight the significant role that renewable energy integration plays in reducing carbon intensity and increasing resource efficiency in apple cultivation. By providing a region-specific perspective on agricultural thermodynamics, the study provides strategic insights into the transition to sustainable and climate-resilient food production systems in Türkiye.

## Introduction

Throughout history, humanity’s wars and harmful industrial activities have led to severe ecological degradation and have accelerated climate change. In particular, the intensive use of coal, petroleum and chemical fertilizers in the agricultural sector increases greenhouse gas emissions, thereby exacerbating global warming^1^. This situation has created significant negative impacts on global food security and crop productivity^2^. In response to the growing population and rising food demand, efforts have been made to enhance crop diversity and yield. These efforts are essential. However, production using unsustainable methods, especially those reliant on fossil fuels, has resulted in serious environmental consequences^3^. In order to ensure a sustainable future, it is crucial to analyse agricultural production systems based on their energy and exergy consumption, as well as their CO_2_ emissions. This is necessary in order to develop environmentally friendly and efficient production strategies^4^.

**Fig. 1:**
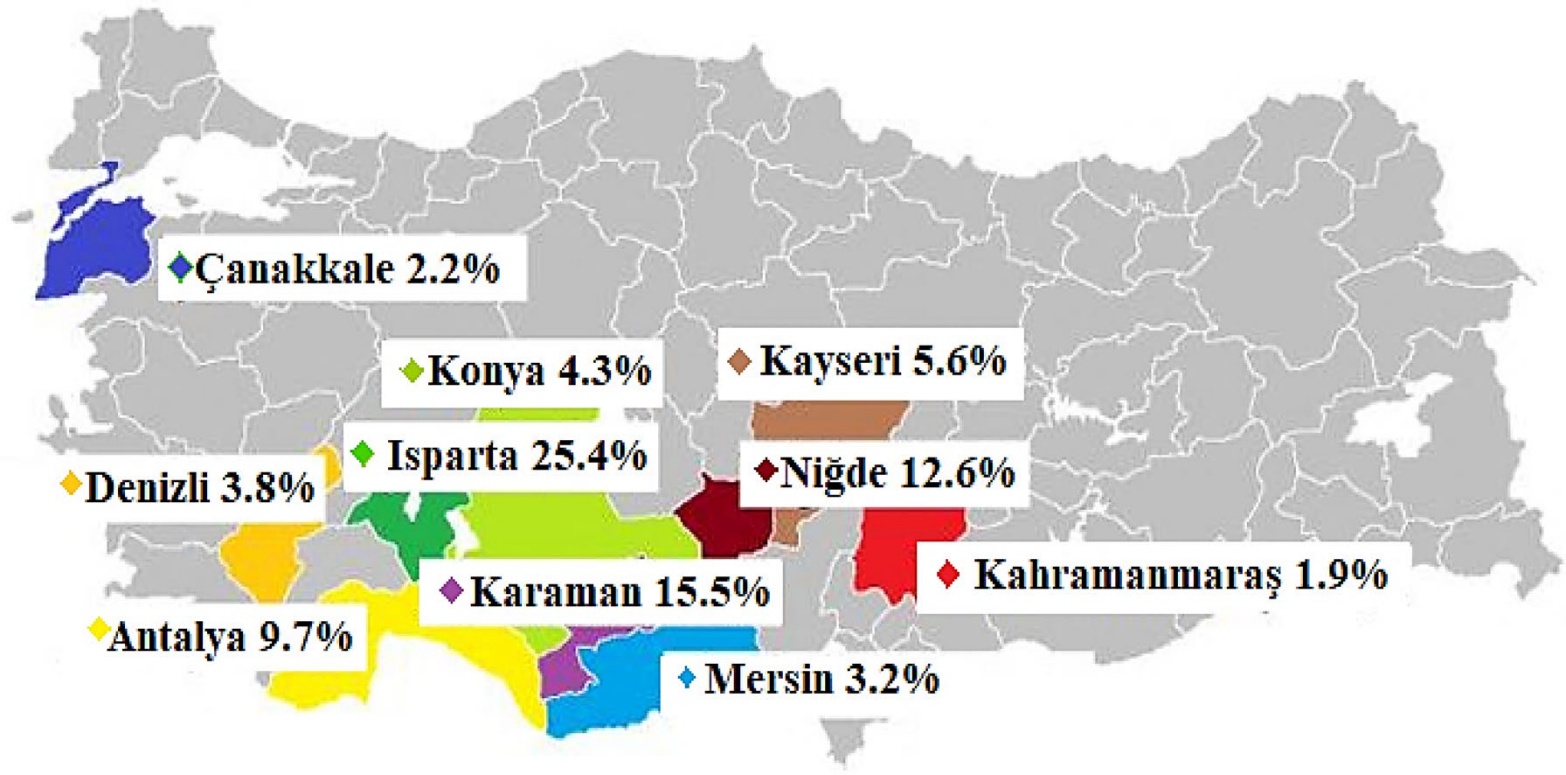
Provinces with the largest share in Türkiye’s apple production^7^.

Apples hold a significant place in human nutrition, being rich in monosaccharides, minerals, dietary fiber, vitamin C and natural antioxidants. Similar to other countries, apples are among the most commonly consumed fruits in Türkiye. China ranks first in global apple production, accounting for approximately 49% of total world output. The country’s annual commercial apple production reaches 24 million tons, of which about 70% is consumed directly, while 30% is used for juice concentrate production^5^. Türkiye is among the leading apple-producing countries worldwide and typically ranks third, following the United States, though its position may vary depending on meteorological conditions. Since 1966, apple production in Türkiye has steadily increased from 440,000 tons to more than 4 million tons today. According to data from the Turkish Statistical Institute (TÜİK), in 2023, 4602.5 tons of apples were produced on 167,4 hectares of land. The total production of fruits, beverages and spice plants in 2024 increased by 2.1% compared to the previous year, with apple production showing a 4.0% rise, indicating a remarkable growth trend^6^. Among Türkiye provinces, Isparta ranks first in apple production with 25.4% of total national output (Fig. 1). Antalya and Niğde also rank among the top producing regions, contributing 9.7% and 12.6%, respectively^7^.

Apple production is significantly influenced by seasonal and environmental factors such as temperature, precipitation and frost. Moreover, the quantity and type of inputs used during production directly affect yield and product quality. Energy consumption, fertilizer and pesticide use and transportation activities define the environmental footprint of production. Therefore, improving energy and exergy efficiency, reducing carbon emissions and developing sustainable production methods in apple cultivation are critical both environmentally and economically. Energy use in agricultural production consists of direct inputs (fuel, electricity) and indirect inputs (fertilizers, pesticides and labor). One of the essential steps in optimizing energy use in crop production is to minimize total energy consumption and identify energy losses. However, energy analysis only measures the amount of energy consumed without considering its quality. In contrast, exergy analysis evaluates the quality of each input by quantifying the potential for useful work and identifying where energy degradation occurs^8,9^. Through this approach, the inputs responsible for the highest exergy losses can be identified, allowing process optimization and improved resource efficiency.

Solar energy can be utilized in agriculture through both stand alone and integrated systems. It reduces air pollution, lowers costs and enhances energy self-sufficiency. Moreover, it can be used to generate electricity for agricultural lands, greenhouses and livestock facilities^10^. Agrivoltaic Systems (AVS) integrate photovoltaic (PV) energy generation with crop production within the same land area. PV panels, typically installed at a height of 2–6 m and spaced approximately 6 m apart, allow sufficient sunlight to reach the crops below^11^. AVS enables simultaneous food and energy production, reduces water consumption and increases land use efficiency. Although shading from PV modules can limit crop yield, this effect can be minimized under optimal microclimatic conditions. Studies have reported that integrated food energy systems can enhance overall land productivity by up to 70%^12^.

Amaducci et al. (2018) demonstrated that AVS applications in biogas maize cultivation achieved a higher Land Equivalent Ratio (LER > 1) compared to conventional PV systems and generated up to twice the energy per unit area^13^. Kadowaki et al. (2012) improved crop yield by installing zigzag PV panels on 12% of greenhouse roof areas^14^. Maia (2020) reported that sheep grazing integrated PV systems generated 5.2 kWh of electricity annually, reduced CO_2_ emissions by 2.8 tons per year and saved USD 698 in costs^15^. Leon (2018) noted that PV greenhouses emit 37% less greenhouse gases than conventional grid connected ones^16^. Giri (2023) calculated a LER of 1.73 and a Payback Period (PBP) of 9.49 years for AVS applications in India^17^. Trommsdorff (2023) reported that in apple cultivation, AVS integration reduced investment costs by 26% and total production costs by 5%, although shading effects caused a 9% decline in annual revenue^18^.

This study is the first integrated thermodynamic and environmental assessment of apple production in Türkiye. Unlike previous research that focused only on energy use, this work simultaneously analyses cumulative energy consumption (CEnC), cumulative exergy consumption (CExC) and cumulative carbon dioxide emissions (CCO_2_E) for three major production regions. It also provides the first regional comparison of cumulative degree of perfection (CDP) and Renewability Index (RI) indicators, revealing fossil fuel dependency and exergy destruction at a system level. Additionally, this is the first study to evaluate the thermodynamic improvement potential of AVS in apple farming. Together, these contributions fill a clear research gap and offer a novel, region-specific roadmap for sustainable agricultural production.

## Methodology

The main objective of energy analysis in agricultural production systems is to minimize input use and improve energy efficiency. Such analyses provide insights into more efficient resource utilization and identify stages where energy consumption can be minimized for maximum yield, thus supporting sustainable production management^19^. In this study, cumulative energy, exergy and CO_2_ consumption analyses were conducted for the apple production processes of three major provinces in Türkiye with high production capacity (Antalya, Isparta and Niğde). Based on these results, sustainability metrics such as CDP and RI were also calculated and an additional scenario analysis was performed for AVS integration to assess possible improvements.

Table 1 presents the amount of inputs used for producing one metric ton (1000 kg) of apples in the three provinces. The data used in the study were obtained through structured, face to face surveys conducted with local apple producers in each region (Antalya, Isparta, and Niğde). The survey included standardized questions on fertilizer amounts, chemicals, diesel consumption, electricity consumption, irrigation water use and cultivation practices. It was also observed that the same survey form and the same set of questions were used in all three regions and that the assumptions and data structure were fully comparable. While the regional input data shown in Table 1 draws from previous regional studies, it has been standardized for system boundaries using a metric ton as the functional unit to ensure comparability across the three regions. Specifically, these input sources are based on comparable methods and a common focus on input-output analysis per unit area. In Antalya, nitrogen and phosphorus use was relatively high, while in Isparta, all fertilizer inputs were comparatively low. Niğde showed high nitrogen, phosphorus and potassium use, indicating greater soil nutrient demand in the region. Similarly, the higher chemical input values in Niğde point to more intensive agricultural practices. Resource use analysis revealed that Antalya had higher electricity consumption, whereas Niğde had the highest diesel use. The most notable difference was in irrigation water use, where Isparta and Niğde consumed nearly 20 times more water than Antalya. These findings indicate that regional climatic conditions, soil characteristics and agricultural practices directly influence resource consumption in apple production.

**Table 1:** Input and output values for production of one ton apple.

Inputs		Unit	Quantity per ton for one ton apple		
			Antalya^20^	Isparta^21^	Niğde^22^
Fertilizers	Nitrogen (N)	kg	9.58	1.99	6.23
	Phosphorus (P_2_O_5_*)*	kg	4.27	0.85	7.12
	Potassium (K_2_O)	kg	3.65	3.74	7.11
	Manure	kg	27.27	-	24.66
Chemicals	Insecticides	kg	0.08	0.15	0.97
	Fungicides	kg	0.95	0.18	1.19
	Herbicides	kg	-	0.13	0.01
Diesel		kg	1.16	2.63	29.91
Electricity		MJ	140.18	52.83	116.36
Water for irrigation		m^3^	5.75	110.07	109.24

Table 2 presents the specific CEnC, CExC and CCO_2_E values of the main inputs used in apple production. The data highlight significant variations in the energy and environmental impacts of different inputs. Among fertilizers, nitrogen fertilizer had the highest specific energy and exergy values (78.2 and 32.7 MJ/kg respectively), indicating its substantial contribution to energy consumption during production and application. In terms of carbon emissions, nitrogen fertilizer also had a high emission factor of 0.09 kg CO_2_/kg. Potassium and phosphorus fertilizers exhibited lower energy and exergy values; however, potassium had a relatively high carbon emission of 0.51 kg CO_2_/kg. In the chemical group, insecticides, fungicides and herbicides had notably high specific energy values (198.8 MJ/kg), revealing their energy-intensive production and application stages. Moreover, herbicides recorded the highest emission rate at 6.3 kg CO_2_/kg. Diesel (57.5 MJ/kg) and electricity (1 MJ/MJ) were the most significant direct energy sources, with diesel showing a high carbon emission value of 0.94 kg CO_2_/kg. Irrigation water, with very low energy and exergy values (0.00102 MJ/kg and 0.00425 MJ/kg), contributed minimally to the system’s total energy balance. Overall, nitrogen fertilizers, pesticides and diesel emerged as the main factors increasing environmental burden in terms of both energy consumption and greenhouse gas emissions.

**Table 2:** Specific energy, exergy and CO_2_ emission values ​​for each input in apple production.

Inputs	Specific CEnC	Specific CExC	Specific CCO_2_E
Fertilizers			
Nitrogen (N)	78.2 MJ/kg^23^	32.7 MJ/kg^24^	0.09 kg/kg^25^
Phosphorus (P_2_O_5_)	17.5 MJ/kg^24^	7.52 MJ/kg^26^	0.15 kg/kg^25^
Potassium (K_2_O)	13.8 MJ/kg^24^	4.56 MJ/kg^27^	0.51 kg/kg^25^
Manure	0.35 MJ/kg^28^	5.33 MJ/kg^29^	0.0462 kg/kg^29^
Chemicals			
Insecticides	198.8 Mj/kg^30^	7.5 MJ/kg^26^	5.1 kg/kg^31^
Fungicides	198.8 Mj/kg^30^	4.6 MJ/kg^27^	3.9 kg/kg^31^
Herbicides	198.8 Mj/kg^30^	32.7 Mj/kg^32^	6.3 kg/kg^31^
Diesel	57.5 Mj/kg^30^	53.2 Mj/kg^24^	0.94 kg/kg^31^
Electricity	1 MJ/MJ^24^	4.17 MJ/MJ^24^	0.14 kg/MJ^33^
Water for irrigation	0.00102 MJ/kg^29^	0.00425 MJ/kg^29^	0.000595 kg/kg^29^

**Fig. 2:**
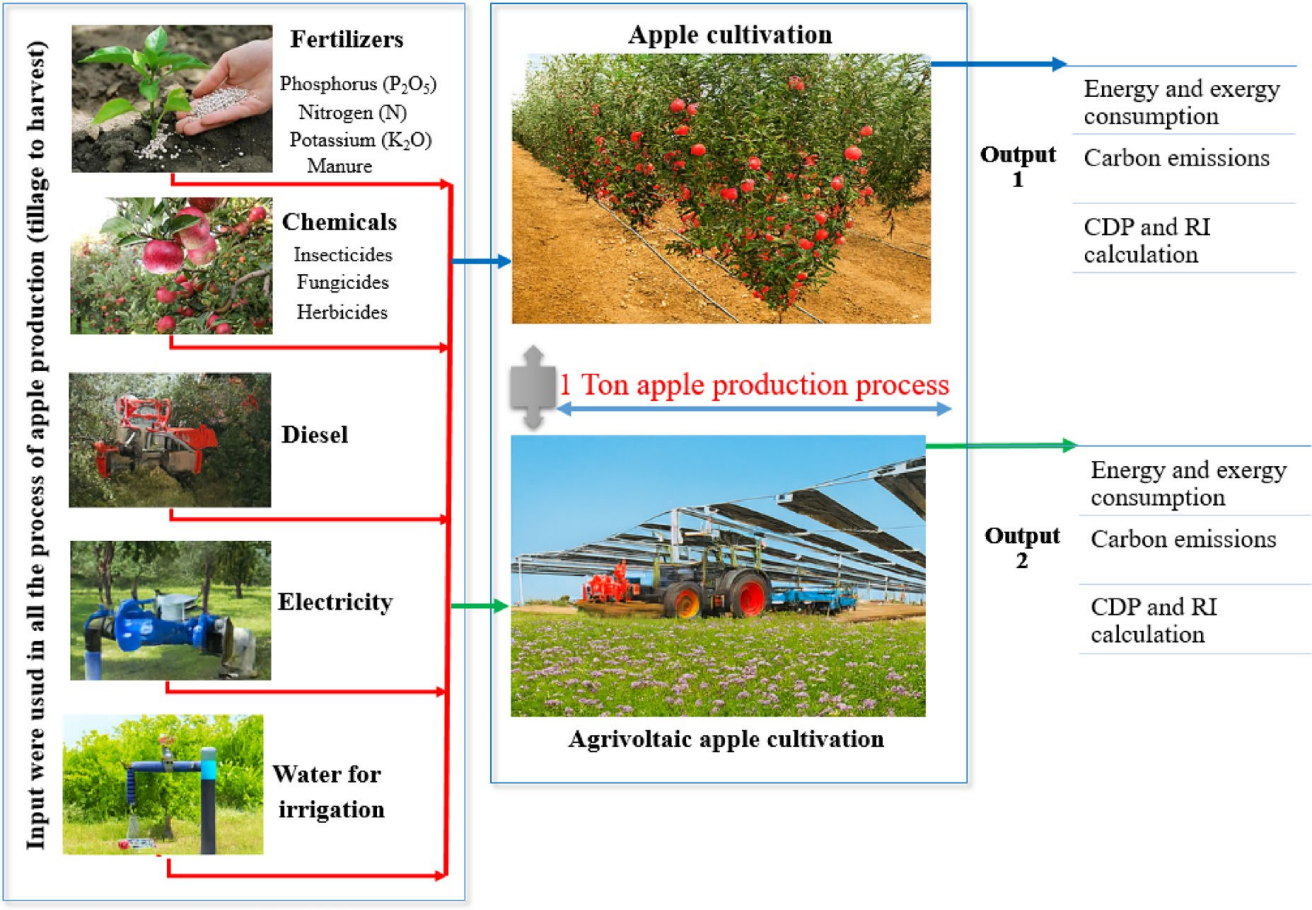
System boundaries and main inputs defined for apple production.

The key input flows required for producing one ton of apples are systematically illustrated in Fig. 2. Fertilizers, chemicals, manure, diesel, electricity and irrigation water represent the fundamental inputs in the production process. While these inputs improve yield, they simultaneously influence total energy consumption, exergy losses and carbon emissions. Hence, the quantity and type of inputs used in apple cultivation are critical determinants of environmental sustainability. The figure emphasizes that agricultural production systems must be evaluated not only from a biological perspective but also through their energetic and environmental dimensions.

In the energy and exergy analyses of the apple production process, mass, energy, exergy and entropy balances were applied. Mass balance ensures equality between the input and output masses, while energy balance is calculated through enthalpy, work and heat interactions^34^. Exergy balance determines system losses by considering environmental and source temperatures and entropy balance reveals the generation of disorder within the system. The exergy flow in production consists of chemical and physical exergy components. System efficiency is evaluated using the CDP, defined as the ratio of the chemical exergy of the product to the total input exergy^24^. Additionally, the cumulative net exergy consumption (CNEx) and the restoration work (Wr) required to offset environmental impacts are calculated^35^.

1 $$\:{\Sigma\:}\left(m\right)\text{i}\text{n}\:=\:{\Sigma\:}\left(m\right)\text{o}\text{u}\text{t}$$

2 $$\sum {\left( {mh} \right)_{{in}} } - \sum {\left( {mh} \right)_{{out~~}} } = Q - {\text{ }}W$$

3 $$\sum {\left( {mb} \right)_{{in}} } - \sum {\left( {mb} \right)_{{out}} } + ~\sum {\left( {1 - \frac{{T_{o} }}{{T_{k} }}} \right)Q_{k} ~ - W} ~ = {\text{ }}X_{{Ioss}}$$

4 $$\sum {S_{{generation}} } = \sum {\left( {ms} \right)_{{out~}} } - \sum {\left( {ms} \right)_{{in}} } - ~\sum {\frac{{Q_{k} }}{{T_{k} }}}$$

5 $$b = b^{{ch}} + {\text{ }}b^{{th}}$$

6 $$b^{{th}} = R_{u} T_{{0_{i} }} y_{i} \ln \left( {y_{i} } \right)$$

7 $$\:CDP=\frac{{\left(\text{m}\text{b}\right)}_{\text{p}\text{r}\text{o}\text{d}\text{u}\text{c}\text{t}}}{\sum\:{\left(m\text{C}\text{E}\text{x}\text{C}\right)}_{\text{r}\text{a}\text{w}\:\text{m}\text{a}\text{t}\text{e}\text{r}\text{i}\text{a}\text{l}\text{s}\:}+\sum\:{\left(m\text{C}\text{E}\text{x}\text{C}\right)}_{\text{f}\text{u}\text{e}\text{l}\text{s}\:}}$$

8 $$\:CNEx=CExC-{Ex}_{p}$$

9 $$\:{W}_{r}={CNEx}_{p}-{CNEx}_{waste}$$

10 $$\:RI=\frac{{W}_{P}-{W}_{r}}{{W}_{p}}$$

Two key sustainability indicators were used to assess system performance. The first, the CDP, represents the ratio of the product’s chemical exergy to the total natural resource exergy consumed during production. A higher CDP indicates more efficient and resource-friendly production. The second, the RI, reflects the environmental impact and renewable character of the system. A positive RI indicates a production process based on renewable and sustainable inputs, whereas a negative RI denotes a dependence on fossil fuels and increased environmental burden^35^.

### Agrivoltaic systems (AVS)

PV systems that generate electricity based on site specific solar irradiation and slope characteristics^36^ can be integrated with agricultural production to form AVS, enhancing both energy and food productivity. The growing demand for energy and food has intensified the need for AVS, which allow simultaneous agricultural and PV energy production. Research has shown that AVS can increase the economic value of agricultural land by more than 30% while minimizing crop yield losses. Furthermore, converting large agricultural areas to AVS installations presents significant potential for expanding national PV energy capacity. For instance, in the United States, transforming lettuce cultivation areas into AVS installations added between 40 and 70 GW of additional capacity. In water-scarce regions, allocating part of agricultural land for solar energy production enhances agricultural security and provides water and economic benefits, even with some trade-offs in food output^37^.

The first AVS prototype, developed in Japan in 2004, has since expanded to over 1,000 operational sites across the country. Studies show that AVS systems can increase land productivity by 35–73% through optimized modeling of plant growth and power generation beneath PV panels^38^. With appropriate PV system design and crop planning, soil productivity can be improved by 60–70%^11^. These findings underscore that AVS offer a sustainable and efficient solution for producing both renewable energy and food, while alleviating land use conflicts and improving climate resilience.

## Results and discussion

This study comprehensively evaluates the energy, exergy and environmental performance of apple production processes conducted in Antalya, Isparta and Niğde provinces. The analysis integrates CEnC, CExC and CCO_2_E to assess the sustainability of resource use and environmental impacts in agricultural production systems. Additionally, exergy based sustainability indicators, namely CDP and RI, were calculated, providing a new perspective by examining their potential improvement through AVS integration. This approach allows for a reduction in exergy losses and an evaluation of the systemic changes in the overall production process. The analyses were carried out using one ton of apple production as the functional unit, considering all major inputs (fertilizers, chemicals, diesel, electricity and irrigation water) from tillage to harvest, while excluding post-harvest and storage stages. The computational framework adopted follows widely recognized methodologies in the literature for energy and exergy analyses. Cumulative energy and exergy values were calculated using the unit energy content and efficiency coefficients of each input and the results were expressed in megajoules (MJ per ton of product). Carbon emission results were reported in kilograms of CO_2_ per ton of product.

The findings reveal that the dynamics of energy use and environmental sustainability vary significantly across Türkiye’s diverse climatic zones. Moreover, the study benchmarks the overall performance of apple production systems against similar agricultural systems reported in the literature. Therefore, this comprehensive energy, exergy and carbon based assessment underscores that regional differences play a crucial role in understanding the environmental performance of agricultural production systems, highlighting the need for sustainable agricultural policies supported by localized planning strategies.

### Cumulative energy consumption in apple production

**Fig. 3:**
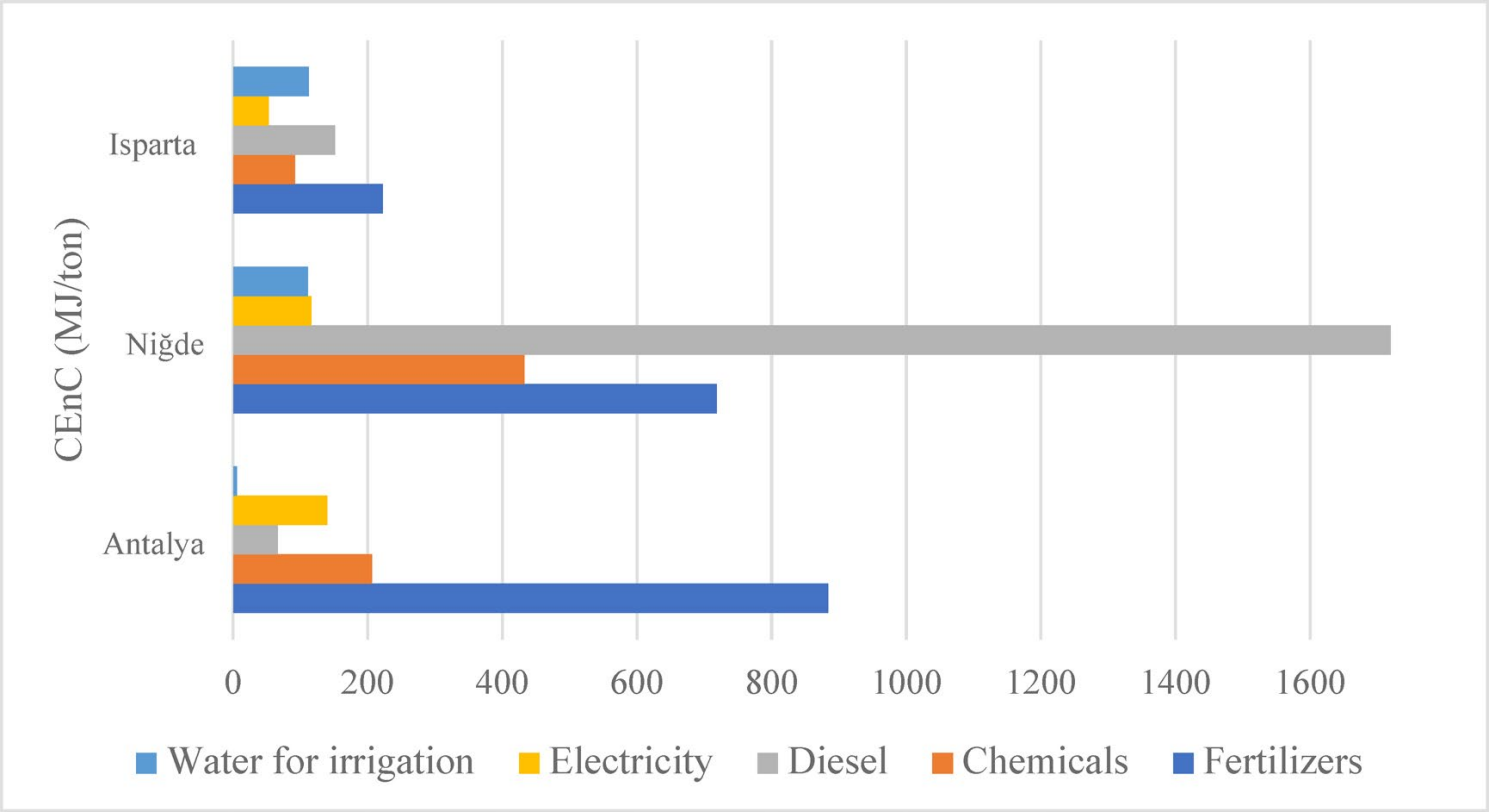
Cumulative energy consumption of different provinces for 1 ton of apple production.

The cumulative energy consumption for various inputs used in apple production in Antalya, Isparta and Niğde is presented in Fig. 3. The data show distinct regional differences, with Niğde exhibiting the highest total energy use at 3098.40 MJ/ton, indicating a more energy intensive production structure compared to Isparta and Antalya.

In Niğde, the majority of total energy input arises from diesel fuel (1719.68 MJ/ton) and fertilizer use (718.22 MJ/ton). These values are significantly higher than those observed in Antalya (66.69 MJ/ton) and Isparta (151.26 MJ/ton) for diesel consumption. This substantial difference can be attributed to the region’s high level of mechanization, the frequent use of mechanical operations and its reliance on diesel powered irrigation systems. Consistent with this, Hesampour et al. (2022) reported that diesel consumption constitutes the dominant share of total energy use in agricultural regions where water extraction and irrigation rely on diesel pumps^39^. Similarly, the high fertilizer consumption in Niğde is linked to the naturally low nutrient content of local soils, prompting farmers to apply more chemical and organic fertilizers to maintain yields. Niğde also demonstrates a significantly higher irrigation energy demand (111.43 MJ/ton) nearly 20 times greater than Antalya (5.87 MJ/ton) reflecting the region’s water scarcity and the energy required for deep groundwater extraction. Juárez-Hernández et al. (2019) observed comparable increases in energy use in irrigation intensive regions^40^.

In Antalya, the total cumulative energy consumption was 1303.11 MJ per ton of apples, with fertilizer use accounting for the largest share at 883.81 MJ/ton. The region applies intensive fertilization practices to achieve high yields, which significantly contributes to total energy consumption. Similarly, in a study analysing the apple production process in Iran, it was observed that replacing chemical fertilizers with farmyard manure reduced overall energy consumption^41^. This can be attributed to the higher energy coefficients of chemical fertilizers compared to organic alternatives. In contrast, diesel and irrigation water consumption in Antalya are relatively low, indicating the widespread use of modern irrigation infrastructure, such as drip irrigation systems. Electricity consumption (140.18 MJ/ton) is slightly higher than in Niğde but shows a balanced distribution within the overall energy profile. The lower diesel (66.69 MJ/ton) and chemical input (206.56 MJ/ton) values compared to Niğde suggest that production processes in Antalya rely on more modern and controlled agricultural inputs.

Isparta, on the other hand, demonstrates a balanced energy consumption profile overall. Both fertilizer and chemical input use are lower than in Antalya and Niğde, while diesel consumption is significantly lower than in Niğde. Additionally, Isparta exhibits the lowest electricity consumption among the three provinces. However, its water consumption is similar to Niğde’s but more than twenty times higher than Antalya’s, highlighting the regional variations in irrigation requirements. This suggests that Isparta employs optimized production techniques for most inputs, except for water use, which remains relatively high due to climatic and geographical factors. The results show that diesel fuel and fertilizers are the dominant contributors to total energy use, while sustainable irrigation systems, particularly those implemented in Antalya, help reduce overall energy demand. These findings indicate that in energy intensive regions such as Niğde, the adoption of solar powered pumping systems could significantly reduce diesel and electricity consumption. Such integration would improve energy efficiency, reduce carbon emissions and ultimately contribute to a more environmentally friendly and sustainable agricultural production process.

### Cumulative exergy consumption analysis in apple production

**Fig. 4:**
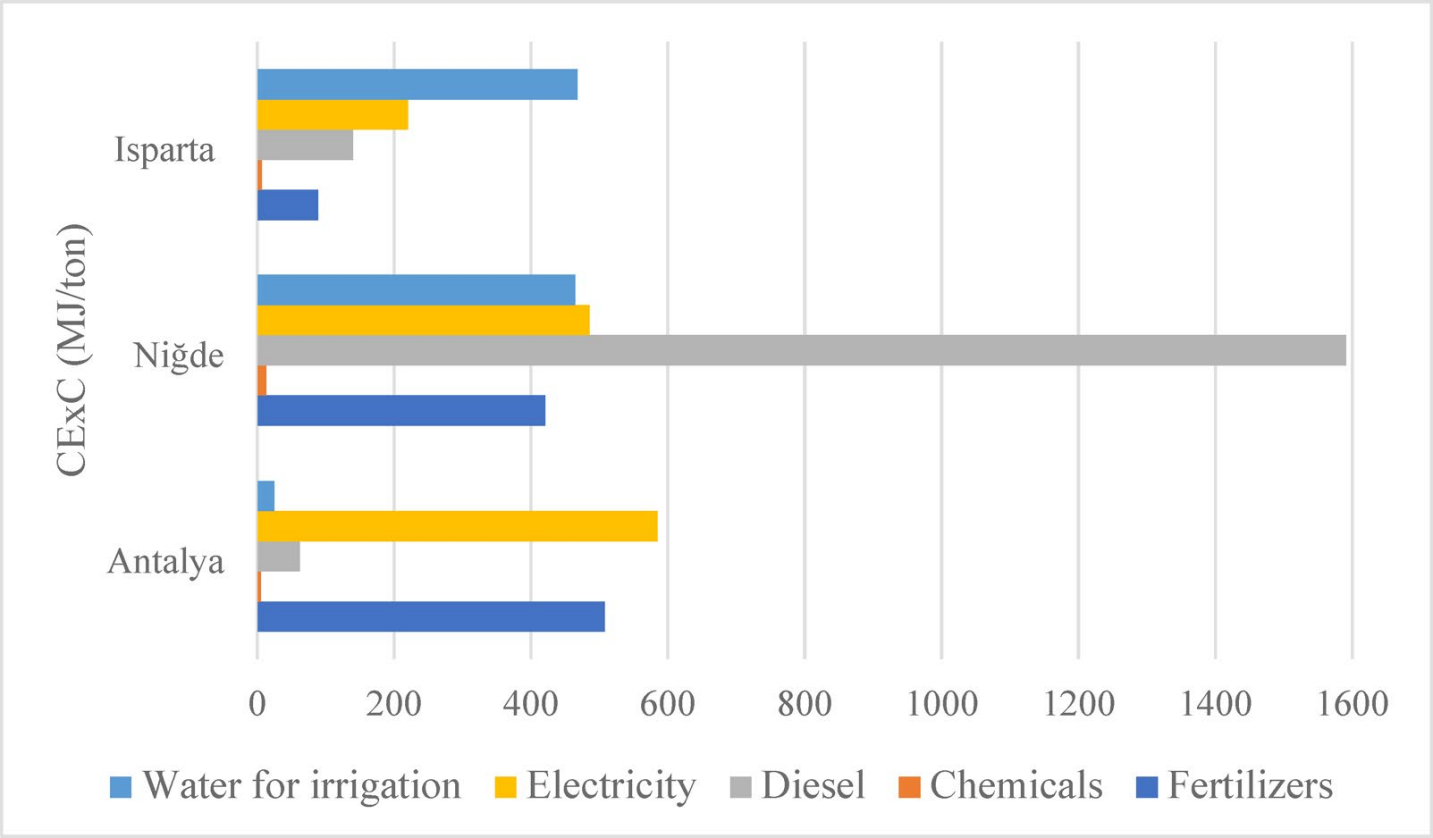
Distribution of cumulative exergy consumption for 1 ton of apple production across provinces.

Figure 4 presents the calculated CExC values for one ton of apple production in Antalya, Isparta and Niğde. The CExC represents the total sum of the exergy equivalents of all energy inputs used in the production process including electricity, diesel fuel, fertilizers, chemicals and irrigation water. Unlike energy analysis, the exergy approach not only quantifies the amount of energy used but also considers energy quality, thereby providing a more comprehensive thermodynamic evaluation of the system’s efficiency.

Among the studied regions, the highest CExC was observed in Niğde (2974.79 MJ/ton), which is considerably higher than that of Antalya (1183.07 MJ/ton) and Isparta (922.77 MJ/ton). This elevated CExC value in Niğde is comparable to those reported in conventional agricultural systems, indicating a relatively inefficient energy use pattern. For instance, in a similar study conducted in Iran, the CExC value for traditional apple production was determined to be 3023 MJ/ton^41^. A substantial portion of Niğde’s exergy consumption originates from diesel fuel (1591.08 MJ/ton), reflecting the region’s dependence on mechanized farming, diesel powered irrigation pumps and water intensive irrigation practices. Consistent findings were reported by Hesampour et al. (2022), who noted that in date production systems, diesel consumption represented the dominant share of total exergy use^39^. Likewise, high diesel fuel consumption has also been documented in the production of various rice cultivars^8^.

In Antalya, the dominant contributors to exergy consumption are electricity (584.56 MJ/ton) and fertilizers (507.37 MJ/ton). Similar to its energy consumption profile, both electricity and fertilizer use exhibit relatively high exergy values. This finding emphasizes the need for more efficient fertilization techniques and the substitution of chemical fertilizers with farmyard manure to enhance system sustainability. The relatively high electricity consumption indicates the widespread use of electrically powered irrigation systems, such as pressurized drip irrigation. Conversely, the exergy values for diesel and irrigation water are notably low, reflecting the reduced demand for both due to improved water efficiency. The exergy contribution of chemical inputs in Antalya is only 4.99 MJ/ton, rendering it negligible within the total exergy balance.

In Isparta, while the total exergy consumption remains relatively low, irrigation water (467.80 MJ/ton) and electricity (220.32 MJ/ton) represent significant shares of total input exergy. This can be attributed to the region’s climatic conditions, which necessitate higher water use and pumping energy during irrigation. However, the limited reliance on fossil fuel-based inputs compared to other regions indicates that Isparta maintains a more balanced and efficient exergy profile overall. These findings reveal substantial differences in energy quality and thermodynamic efficiency among regional apple production systems. Niğde, in particular, demonstrates a lower energy conversion efficiency, highlighting the need for targeted improvements. The implementation of solar-powered pumping systems, soil-specific irrigation strategies and optimized fertilization practices could significantly enhance exergy efficiency while simultaneously strengthening the overall sustainability of agricultural production system.

### Cumulative carbon emission analysis in apple production

**Fig. 5:**
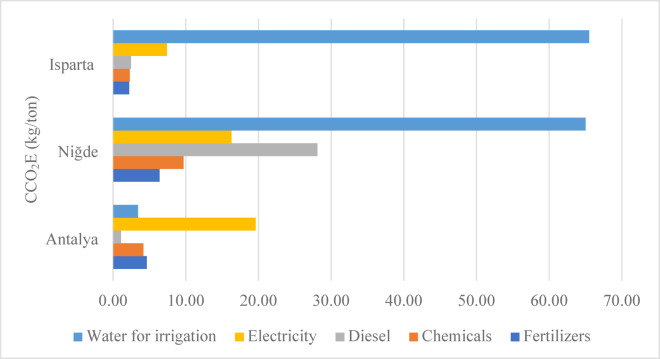
Cumulative CO_2_ emissions associated with 1 ton of apple production.

The calculated CCO_2_E values for the production of one ton of apples in Antalya, Isparta and Niğde are presented in Fig. 5. This indicator encompasses the total CO_2_ equivalent emissions generated from all energy inputs used throughout the production process. The carbon emission levels vary significantly depending on the intensity of agricultural mechanization, irrigation practices, electricity consumption and chemical input usage across the three provinces.

The highest total carbon emission was recorded in Niğde at 125.48 kg CO_2_/ton, substantially exceeding the corresponding values for Isparta (79.87 kg CO_2_/ton) and Antalya (32.92 kg CO_2_/ton). This elevated emission in Niğde is primarily attributed to the extensive use of diesel fuel (28.11 kg CO_2_/ton) and irrigation water (65 kg CO_2_/ton). Such results indicate a heavy dependence on diesel powered deep well pumps and energy intensive irrigation systems due to the region’s arid climate and high water requirements.

In contrast, Antalya exhibited the lowest total emissions (32.91 kg CO_2_/ton). This reduction is mainly due to the Mediterranean climate, which lowers irrigation demands and the greater reliance on electricity instead of diesel fuel. Although electricity-related emissions (19.63 kg CO_2_/ton) slightly exceed those from diesel, the overall emissions remain considerably lower than in other regions. Moreover, the lower use of fertilizers and chemical inputs further contributes to Antalya’s superior environmental performance.

Isparta demonstrates intermediate emission levels, higher than Antalya but lower than Niğde. The CO_2_ emissions from irrigation water reach 65.49 kg CO_2_/ton, accounting for approximately 82% of total emissions. The region’s high elevation and sloped terrain necessitate pressurized irrigation systems, which substantially increase energy requirements. Integrating agrivoltaic systems can significantly boost efficiency by supplying renewable energy and cutting fossil fuel dependence. Despite high irrigation related emissions, the diesel and chemical contributions in Isparta remain relatively low, indicating better fuel efficiency.

Comparable findings have been reported in the literature. Yildizhan et al. (2021) identified that manure, electricity and water use were major contributors to CO_2_ emissions in apple production systems in Iran^41^. Similarly, Pelvan and Ozilgen (2017) found that diesel, natural gas and electricity consumption accounted for the largest share of carbon emissions in black tea production^42^. Degerli et al. (2015) reported that in wheat production systems in Turkey and Germany, the main emission sources were fertilization, irrigation and diesel use, with Turkey exhibiting higher CO_2_ emissions due to greater diesel and fertilizer dependency^43^.

A comparative summary of the results can be outlined as follows:

- Niğde: Highest carbon emissions; dominant sources are diesel and irrigation water.
- Isparta: Moderate emissions; primary source is irrigation water.
- Antalya: Lowest emissions; electricity based system with balanced and sustainable agricultural practices.

These findings indicate that in high consumption regions such as Niğde, the implementation of renewable energy assisted irrigation systems (e.g., solar powered pumps) and efficient water management technologies (such as drip irrigation and moisture sensor based automation) can significantly mitigate carbon emissions. The integration of AVS not only enhances renewable energy utilization but also reduces water evaporation through shading, thereby promoting a more efficient and climate resilient agricultural process. In Antalya and Isparta, the existing systems already demonstrate a strong potential for further emission reductions, particularly through the integration of agrivoltaic structures for on-site electricity generation. Overall, the cumulative carbon emission analysis highlights that regional production conditions and energy resource profiles play a decisive role in determining the environmental performance of apple cultivation. Therefore, developing location-specific sustainable strategies tailored to each province’s climatic conditions, water availability and energy infrastructure is of vital importance for achieving low carbon agricultural production in Türkiye.

The findings of this study have important practical implications for Türkiye’s agricultural and energy policies. They will significantly contribute to reducing regional disparities in energy use, exergy destruction and carbon intensity, as well as reducing dependence on fossil fuels, increasing irrigation efficiency and expanding the use of renewable energy in agriculture. Our findings demonstrate that regions with high diesel based irrigation demand, such as Niğde and Isparta, can significantly benefit from solar assisted irrigation technologies and AVS applications. Similarly, the high CDP and RI values ​​obtained in Isparta support the effectiveness of precision agriculture practices and optimized fertilizer management, as part of Türkiye’s sustainable agriculture strategy. Therefore, the thermodynamic data presented in this study provide a guiding framework for policymakers to prioritize renewable energy supported agricultural practices, design region specific support mechanisms and accelerate the national transformation toward resource efficient, climate resilient food production.

### CDP and RI analysis

**Fig. 6:**
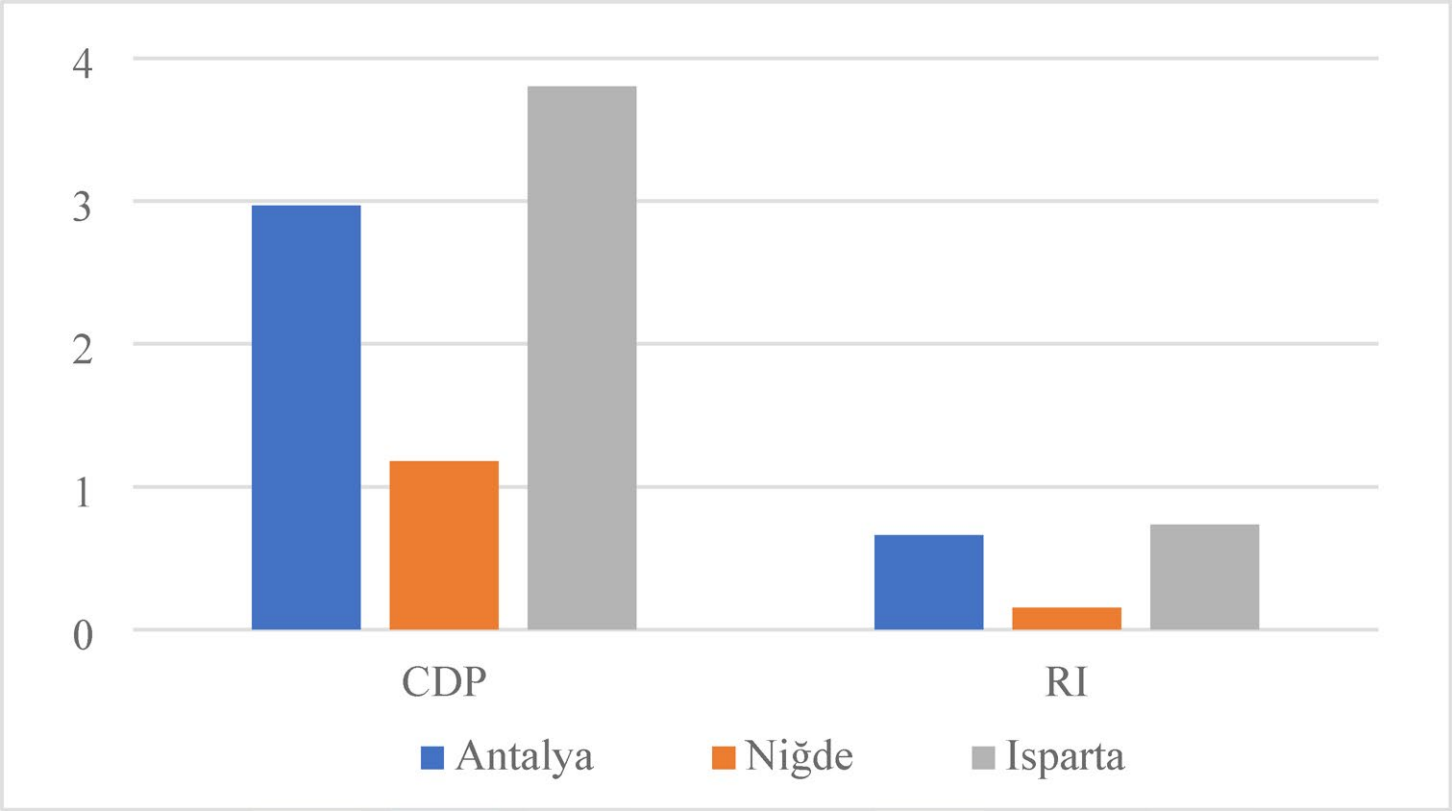
CDP and RI values by provinces.

Figure 6 presents the calculated values of the CDP and RI for the production of one ton of apples in Türkiye’s three major apple-producing provinces: Antalya, Isparta and Niğde. In this study, the chemical exergy flow of the apple product was taken as 3.51 MJ/kg, based on data reported in the literature^41^. These indicators evaluate the thermodynamic sustainability performance of agricultural production systems by considering not only the quantity of energy consumed but also the potential of that energy to be converted into useful work and the degree of renewability of the resources employed.

According to the results obtained, the highest CDP value was determined in Isparta with 3.80, followed by Antalya with 2.97 and Niğde with 1.18. Similarly, the RI values were calculated as 0.74 for Isparta, 0.66 for Antalya and 0.15 for Niğde. These findings indicate that Isparta exhibits the highest thermodynamic efficiency and renewability in apple production, whereas Niğde demonstrates a lower efficiency level and a higher dependence on fossil energy sources. The high CDP and RI values in Isparta can be attributed to the region’s mild climatic conditions and the widespread use of balanced irrigation and fertilization practices. These factors reduce energy and exergy losses, thereby enhancing the overall efficiency of the system. Conversely, Niğde’s lower performance indicators stem from its high electricity consumption due to deep well irrigation, intensive diesel fuel use and the energy intensive nature of agricultural production under continental climatic conditions. Similarly, Taki and Yıldızhan (2018) reported that systems dependent on fossil fuels exhibit significantly lower CDP and RI values^44^. A CDP value of 3.80 in Isparta suggests that exergy destruction in the system is minimal and that a substantial portion of inputs is effectively converted into useful work. In contrast, the CDP value of 1.18 in Niğde indicates significant exergy losses and inefficient energy utilization. This finding is consistent with the results reported by Özilgen and Sorgüven (2011), who observed that fossil fuel based agricultural systems generally demonstrate low thermodynamic performance^45^.

In terms of RI values, positive results were obtained for all provinces (Isparta: 0.74, Antalya: 0.66, Niğde: 0.15), suggesting that all systems are at least partially renewable. However, the higher RI in Isparta indicates that renewable energy inputs in this region more effectively offset fossil based inputs. Furthermore, the low exergy consumption in Isparta is considered one of the key factors behind its higher CDP and RI values. Conversely, Niğde’s low RI value represents the weakest renewability profile among the provinces. Therefore, the adoption of solar-assisted irrigation systems, organic fertilizer use and renewable energy based mechanization should be prioritized in Niğde to improve sustainability performance. Regional climatic differences, irrigation methods and the type of energy sources used have a direct impact on the thermodynamic sustainability of apple production. Accordingly, targeted strategies should be developed for each region. Integrating renewable energy systems, promoting drip irrigation technologies and applying precision agriculture based on optimized inputs can improve CDP and RI values, ultimately making apple production in Türkiye more efficient, low carbon and sustainable.

### Effect of agrivoltaic system integration on thermodynamic sustainability

Increasing energy demand, climate change and the limited availability of arable land have made it essential to develop integrated systems that simultaneously address food and energy production. In this context, AVS offer a sustainable solution by enabling both agricultural and solar energy production on the same land area. These systems enhance land use efficiency, reduce carbon emissions and optimize water use, particularly under arid and semi-arid climatic conditions^11,12^. Agrivoltaics contribute to the development of climate-resilient agriculture, while also supporting energy security, economic stability and environmental sustainability^13,46^.

**Fig. 7:**
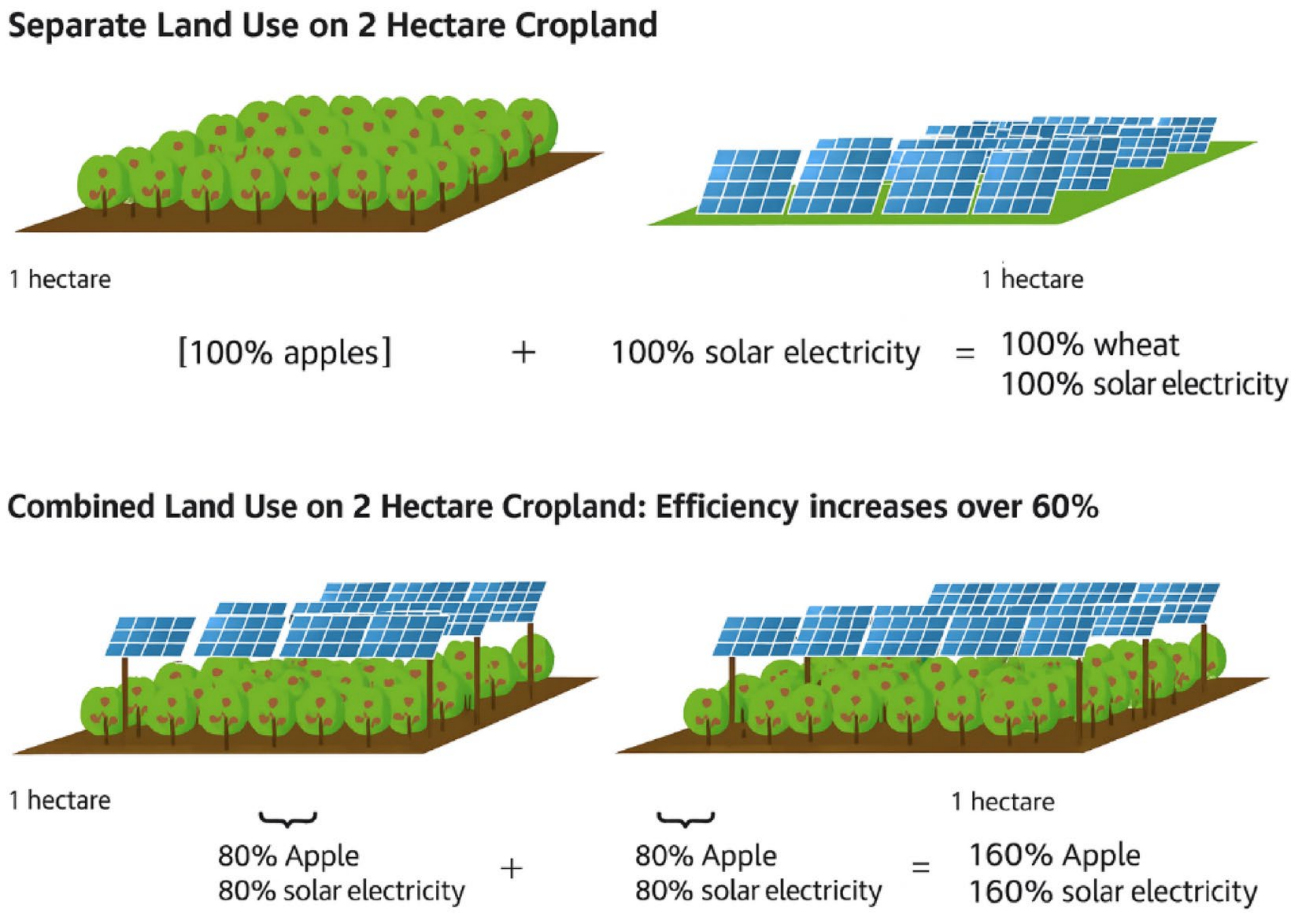
Comparison between conventional agriculture and agrivoltaic systems^47^.

In a system illustrated in Fig. 7, apple trees are integrated beneath PV panels to compare the efficiency of conventional agriculture and AVS based cultivation^47^. As shown, AVS increases LER by combining agricultural and solar energy production on the same plot. Research indicates that, with an appropriate system design, this ratio can exceed 1.0 and the overall productivity can increase by up to 70%^13^. Despite the partial shading effect, changes in microclimatic conditions (such as improved soil moisture retention and reduced ambient temperature) can positively influence plant growth and enhance water use efficiency^48^.

Moreover, studies on apple cultivation have shown that AVS structures can also protect crops from hail damage, providing an additional benefit beyond energy generation^18^. Therefore, agrivoltaic systems represent an effective approach to simultaneously improve agricultural productivity and renewable energy output, particularly in arid and semi-arid regions.

**Fig. 8:**
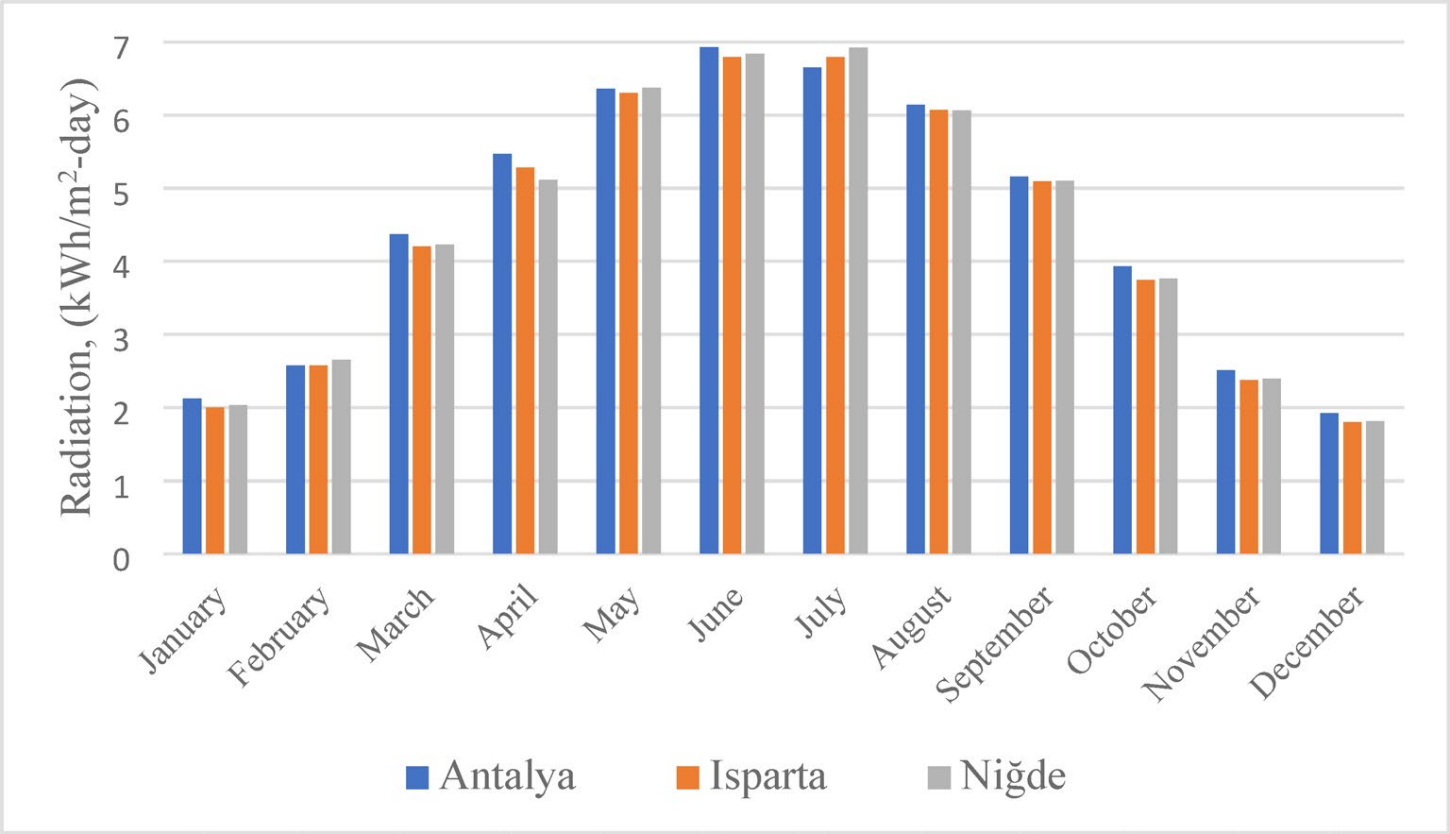
Monthly solar radiation values for the provinces of Antalya, Isparta and Niğde^49^.

As shown in Fig. 8, the annual average solar radiation values of the three provinces (Antalya (4.51 kWh/m²-day), Isparta (4.42 kWh/m²-day) and Niğde (4.44 kWh/m²-day)) are relatively high and very close to each other^49^. There is a direct relationship between high solar radiation levels and the efficiency of AVS. In regions with higher radiation, both greater solar energy generation and reduced water evaporation due to shading contribute to improved agricultural productivity^10,38^. Therefore, Türkiye’s diverse climatic regions offer significant potential for variety, optimization and site specific design in agrivoltaic system applications.

**Fig. 9:**
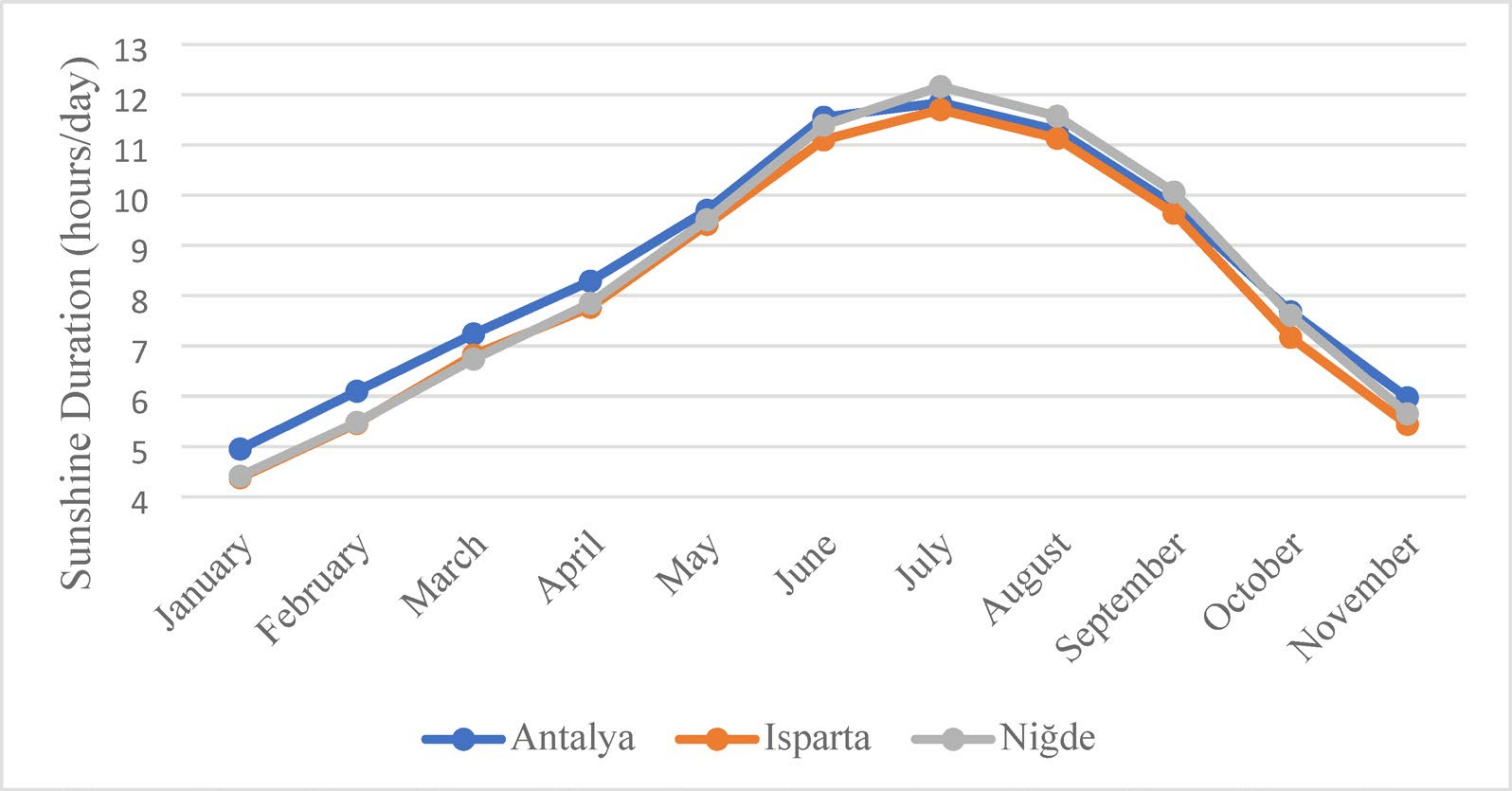
Sunshine duration by months in Antalya, Isparta and Niğde^49^.

A comparative analysis of sunshine duration across Antalya, Isparta and Niğde is presented in Fig. 9. Antalya exhibits the highest solar potential, with an annual sunshine duration of approximately 3011 h, followed by Niğde (2930 h) and Isparta (2858 h)^49^. These data support the feasibility of implementing solar powered irrigation systems, particularly in semi-arid regions such as Niğde and Isparta. Longer sunshine duration not only enhances the energy generation potential of PV panels but also contributes to reduced crop water demand and improved soil moisture retention through the shading effect^50^. Consequently, agrivoltaic systems provide significant environmental benefits, including water conservation and reduction of carbon emissions, while simultaneously producing renewable energy^16,18^.

AVS offer a strategic solution for countries like Türkiye, which have diverse climatic regions, by enhancing both energy production and food security. Furthermore, studies indicate that AVSs can:

- Reduce CO_2_ emissions by 30–40%^16^,
- Lower irrigation requirements by up to 30%^48^.
- Increase the economic value of farms by more than 30%^46^.

Beyond these advantages, the literature shows that AVS can reduce investment costs by 26% and total production costs by 5%^18^. Moreover, viable Payback Periods (PBP) of 9.49 years have been calculated for similar AVS applications^17^. These findings reveal that AVSs present a feasible, environmentally friendly and economically beneficial model for agricultural production in Türkiye. Furthermore, AVS integration allows for the management of economic risks based on regional differences in the agricultural sector. It directly reduces operating costs in regions characterised by fossil fuel dependency and high diesel costs. In areas where high-pressure irrigation systems require energy, AVS balances energy costs with self-consumption. Furthermore, AVS promotes long-term financial sustainability by stabilising electricity consumption and enhancing the economic value of agricultural land.

**Fig. 10:**
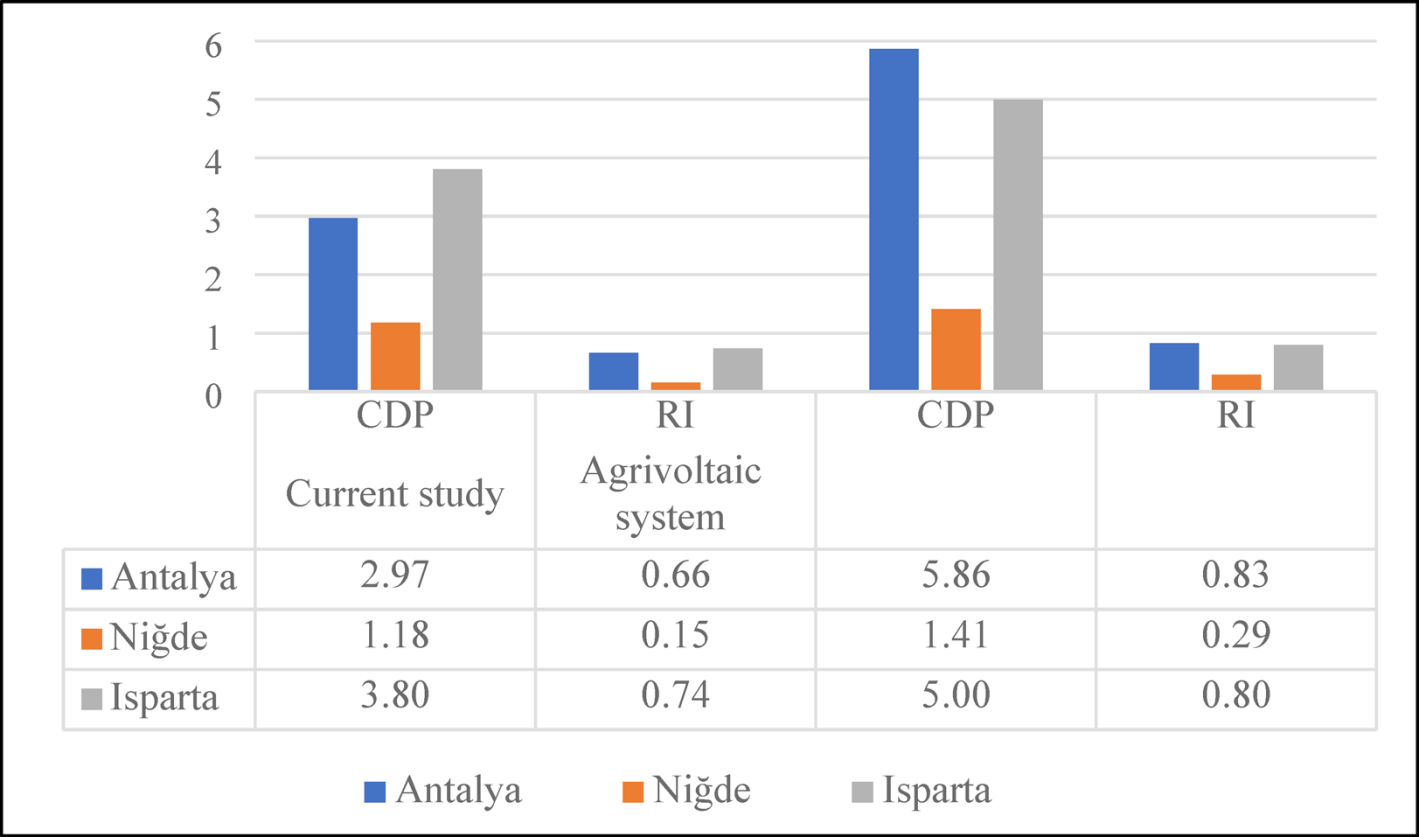
Comparison of CDP and RI values for agrivoltaic and conventional systems.

Figure 10 illustrates how the CDP and RI values for apple production processes in Antalya, Isparta and Niğde change between the current conditions and after AVS integration. The analysis results demonstrate a clear improvement trend in all regions following the implementation of AVSs.

In the current system, the CDP values for Antalya, Isparta and Niğde were calculated as 2.97, 3.80 and 1.18, respectively, while the RI values were 0.66, 0.74 and 0.15. After integrating AVSs, the corresponding values increased to 5.86, 5.00 and 1.41 for CDP and 0.80, 0.83 and 0.29 for RI. These findings clearly indicate that the inclusion of PV energy significantly enhances the system’s overall energy efficiency and renewability.

The increase of the CDP value in Antalya from 2.97 to 5.86 (97% improvement) demonstrates that electricity generation from solar energy substantially reduces exergy losses in the system. Similarly, in Isparta, the CDP value rose from 3.80 to 5.00 (32% increase). The RI values reaching approximately 0.83 for Antalya and 0.80 for Isparta indicate that the production systems have become nearly balanced with renewable components. In contrast, Niğde showed an increase in CDP from 1.18 to 1.41 and RI from 0.15 to 0.29, representing the highest improvement rate (93%) among the regions. This outcome can be attributed to the region’s relatively high electricity consumption, where the use of solar-generated electricity significantly enhanced the system’s renewability. These results demonstrate that AVSs, particularly in irrigation processes with high electricity demand, reduce fossil fuel dependency and contribute to making the agricultural energy chain more sustainable. Additionally, the shading effect of PV panels can improve water use efficiency in hot climates by minimizing evaporation losses. This not only improves energy and exergy performance but also positively influences resource conservation and environmental sustainability. After AVS integration, all three regions exhibited substantial improvements in thermodynamic sustainability indicators, with the highest increase observed in Niğde and the lowest in Isparta. This finding shows that the integration of PV is directly related to regional climatic conditions and energy consumption profiles. Therefore, it is necessary to develop region specific renewable energy strategies across Türkiye.

## Conclusions

This study has demonstrated that the thermodynamic performance and environmental sustainability of apple cultivation vary substantially across Türkiye’s main production regions. Energy, exergy and carbon analyses provided a holistic perspective for evaluating regional farming practices. Among the provinces analysed, Niğde exhibited the highest cumulative energy (3098 MJ/ton) and exergy (2975 MJ/ton) consumptions, driven by intensive diesel-based irrigation and mechanization. This resulted in the largest carbon footprint (125 kg CO_2_/ton), reflecting the region’s dependence on fossil-based energy. In contrast, Antalya presented the lowest total energy use (1301 MJ/ton) and carbon emissions (33 kg C kg CO_2_/ton), highlighting the benefits of electricity dominated systems and efficient irrigation infrastructure. Isparta demonstrated a balanced performance, achieving the highest CDP (3.80) and RI (0.74) values, indicating an advanced level of thermodynamic efficiency and renewability.The integration of AVS into apple farming has shown a transformative impact on sustainability indicators. When PV panels were used to meet electricity demand for irrigation and post-harvest operations, CDP values ​​increased by up to 97%, while RI values ​​improved by approximately 93%. This improvement was most pronounced in Niğde, where fossil energy dependence was greatest. These outcomes reveal the dual advantage of agrivoltaics in reducing exergy destruction and mitigating carbon intensity.

The main drivers of sustainability in apple cultivation are overall regional climatic conditions, irrigation energy sources and fertilizer management. Crucial policy and technological strategies for achieving low carbon and resource efficient production include solar powered pumping, precision nutrient management and efficient irrigation design.

## Data Availability

The authors declare that the data supporting the findings of this study are available within the paper.
